# Exploring drivers of modern Hanfu purchase in digital commerce: A mixed-methods perspective

**DOI:** 10.1371/journal.pone.0337228

**Published:** 2025-11-19

**Authors:** Hao Li, Yucheng Ding, Yuhang Wu, Jiake Ye

**Affiliations:** 1 Silk and Fashion Culture Research Center, Zhejiang Sci-Tech University, Hangzhou, Zhejiang, People's Republic of China; 2 Fashion Design College of Istituto Marangoni Zhejiang Sci-Tech University (International Institute of Fashion Technology), Zhejiang Sci-Tech University, Hangzhou, Zhejiang, People's Republic of China; 3 The College of Theater Film and Television Artistic Design, Sichuan University of Media and Communications, Chengdu, Sichuan, People's Republic of China; 4 College of Intelligence Broadcasting and Communications, Sichuan University of Media and Communications, Chengdu, Sichuan, People's Republic of China; 5 Research Institute of Area and International Communication Studies, Sichuan University of Media and Communications, Chengdu, Sichuan, People's Republic of China; Dong-A University College of Business Administration, KOREA, REPUBLIC OF

## Abstract

The rapid growth of the modern Hanfu market, driven by digital commerce and social media, has increased the interest in understanding consumer purchase intentions. This study investigates the key factors influencing online purchase intentions for modern Hanfu by integrating text mining, grounded theory, and quantitative analyses. A dataset of 5,992 consumer reviews from major Chinese e-commerce platforms was analyzed using natural language processing techniques to extract critical themes. Additionally, a structured survey (n = 344) was conducted to develop and validate a measurement scale for assessing the key determinants of purchase intention. The findings reveal that co-design has the strongest influence on purchase intention (β = 0.252), followed by website attractiveness (β = 0.235) and product quality (β = 0.160). Despite product quality being a dominant theme in consumer reviews, its impact on the quantitative model is moderate (β = 0.160). Social media engagement and cultural identity emerge as significant but less frequently discussed factors, while merchant service has the weakest effect (β = 0.145). This study contributes to the literature by integrating cultural identity and co-design into purchase intention models, offering theoretical insights into heritage-driven fashion consumption. Future research should explore demographic variations, cross-cultural comparisons, and longitudinal trends to further refine our understanding of modern Hanfu purchase behavior. These results suggest that the top priority of modern Hanfu brands in their marketing management activities is to encourage and promote consumer engagement. Optimizing website design, improving product quality, considering social media strategies, tapping into the essence of ethnic culture, and enhancing customer service are also important.

## Introduction

The rapid advancement of digital technologies and social media has positioned the online consumption of fashion products as a critical focus for academia and industry. Online shopping transcends the temporal and spatial constraints of traditional retail and reshapes consumer decision-making through personalized recommendations and virtual “try-on” technologies [1,2]. For fashion products characterized by high perceived risks (e.g., aesthetic mismatches and sizing discrepancies) and emotion-driven attributes (e.g., identity expression and social validation), understanding online consumption behavior is vital [3]. Within this context, purchase intention, as a key antecedent of actual consumption behavior [4], serves as a pivotal entry point for deciphering consumer decision-making mechanisms. Existing literature highlights the multifaceted drivers of purchase intention in the fashion sector, encompassing functional attributes (e.g., quality and price), sociocultural factors (e.g., brand narratives and cultural identity), and contextual elements (e.g., social media engagement and website usability) [5,6]. However, existing literature predominantly focuses on fast fashion or luxury segments, overlooking culturally symbolic products like modern Hanfu. This gap is particularly evident in studies that fail to consider how digital ecosystems shape identity-driven consumption within heritage fashion contexts.

This study focuses on modern Hanfu, a garment category that bridges traditional Chinese attire with modern designs. Defined as a revitalized form of ancient Han Chinese clothing, modern Hanfu retains traditional production techniques (e.g., flat cutting and cross-collar closures), while incorporating modern materials (e.g., polyester blends) and aesthetic motifs to align with contemporary lifestyles [7,8]. By 2023, China’s modern Hanfu market reached ¥14.47 billion (approximately $2 billion), with online sales accounting for 69% (¥10 billion) and a consumer base exceeding 10 million [9]. The sector currently faces three critical challenges: first, balancing the preservation of cultural symbolism (e.g., adherence to dynastic-era tailoring principles and symbolic motifs like dragon-and-phoenix embroidery) with modern aesthetic demands, particularly reconciling historical accuracy in fabric composition (e.g., ramie, silk) and construction techniques (e.g., cross-collar design) with practical wearability for urban lifestyles [7,10–12]; second, addressing the paradox of youth-driven “cultural root-seeking” through modern Hanfu consumption, where it simultaneously functions as a vehicle for neo-Confucian identity reconstruction (e.g., ritualized dressing for traditional festivals) and a commodified form of subcultural capital within digital-native communities [12–14]; and third, overcoming systemic industrial bottlenecks including fragmented artisan production standards (e.g., lack of unified sizing protocols for Ru Qun and Zhi Duo garments), insufficient research and development investment in textile innovation (e.g., moisture-wicking historical fabric recreations), and brand homogenization exacerbated by algorithmic-driven fast-fashion replication on e-commerce platforms [15–17]. These challenges underscore the liminal positioning of modern Hanfu between heritage preservation and digital-era commercialization, highlighting the need to examine how cultural identity is negotiated in online consumer spaces.

Notably, modern Hanfu consumption thrives in social media and e-commerce ecosystems. Young consumers engage with user-generated content to share styling experiences and cultivate subcultural communities, fostering a unique cultural consumption landscape [18–20]. These digital footprints offer rich empirical data for unraveling the mechanisms of purchase intention in culturally symbolic products—a resource yet to be fully exploited. However, the mechanisms through which cultural identity is constructed, negotiated, and transformed into purchase motivation remain underexplored under the background of fashion purchase intention. Furthermore, existing models of fashion purchase intention often adopt a tripartite analytical lens: first, functional drivers emphasize utilitarian attributes such as material durability, cost-performance ratios, and ergonomic design [21,22]; second, psychosocial drivers interrogate identity projection through brand narratives, peer endorsement dynamics, and ideological congruence [23,24], alongside technological drivers evaluating interface optimization and immersive tools like virtual try-on systems [25,26]. However, such studies are predominantly derived from Western contexts, neglecting culturally specific products such as modern Hanfu and failing to interrogate how cultural identity is mediated by social media into purchasing motivations. Further, while existing scales rely predominantly on quantitative metrics that fail to capture semantic nuances in consumer reviews, the systematic analysis of such textual data holds considerable potential for addressing information asymmetry between consumers and sellers, thereby enhancing purchase intentions through improved transparency and trust-building mechanisms [27]. To address these theoretical and methodological gaps, this study proposes a mixed-methods framework that integrates text mining, grounded theory, and structural equation modeling (SEM) to (1) extract salient and latent drivers from modern Hanfu-related online reviews and (2) quantify the structural relationships between social media interactivity, cultural symbolism, and purchase decision pathways.

**Fig 1:**
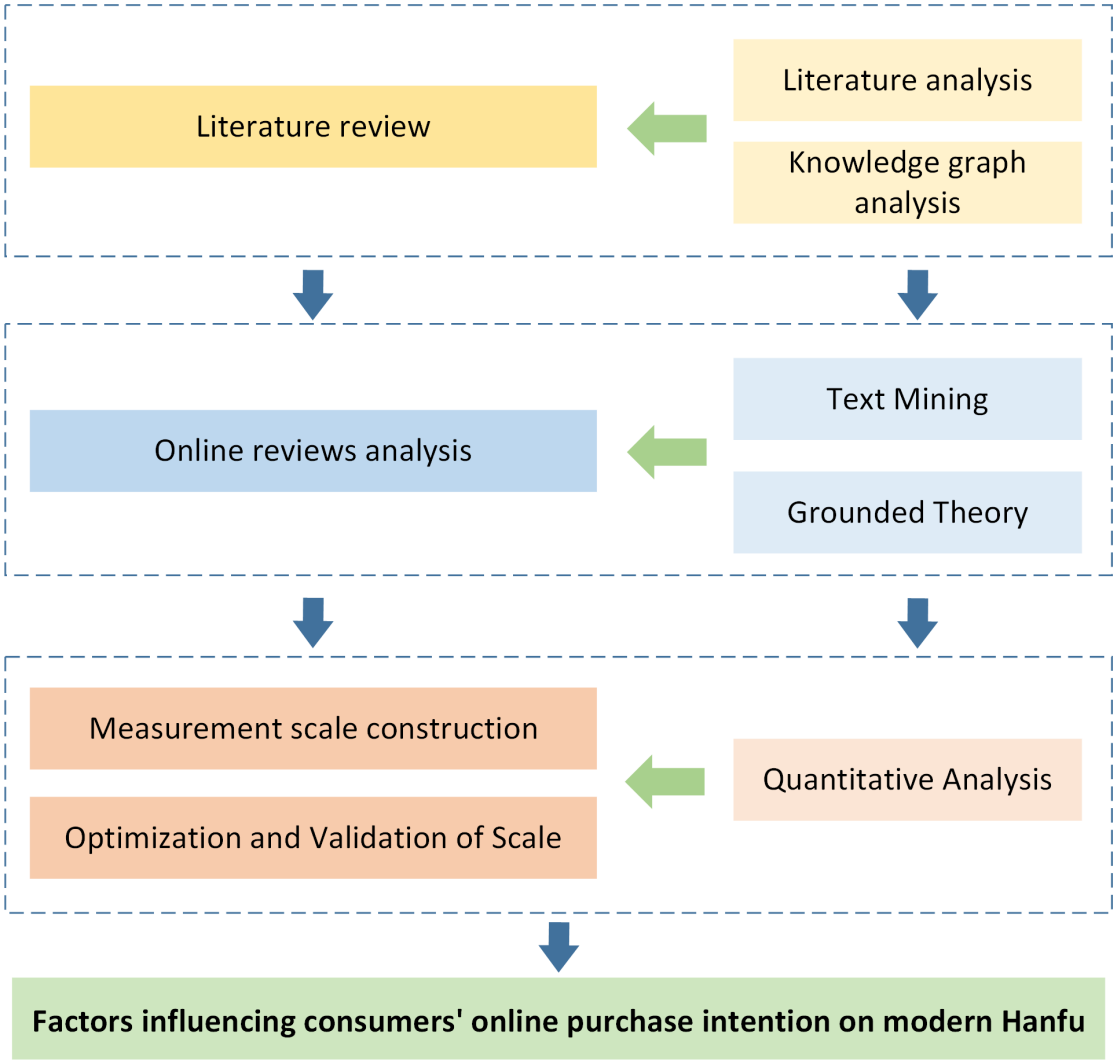
Research road map.

This study expands purchase intention models to culturally symbolic consumption, revealing the dual role of social media in cultural identity formation and commercial conversion. Moreover, this study provides data-driven strategies for modern Hanfu brands to optimize design, marketing, and post-purchase services, facilitating the digital transformation of traditional cultural industries. The research framework illustrated in Fig 1 encompasses data collection, scale development, and model validation aimed at advancing methodological and empirical insights into cultural consumption studies.

## Research background

### Hanfu

#### Cultural significance and identity.

Hanfu represents a significant aspect of Chinese cultural heritage, embodying the historical and philosophical traditions of the Han Chinese people. The importance of Hanfu in reflecting traditional Chinese philosophical culture, including Confucianism, Taoism, and Buddhism, has been emphasized [12]. The revival of Hanfu is a way to reconnect with China’s cultural roots and promote cultural identity among younger generations [28,29].

The concept of modern Hanfu is closely tied to Chinese nationalism, serving as a symbol of cultural pride and identity. The construction of modern Hanfu identity across spatiotemporal scales has been critically examined, particularly regarding its role in contemporary Chinese nationalism [7]. The modern Hanfu movement is a way for young Chinese people to assert their cultural heritage and connect with their national identity.

Modern Hanfu has become a popular element of cultural tourism, offering tourists a unique experience of traditional Chinese culture. The integration of modern Hanfu into tourism destinations enhances the cultural experience; for example, Zhong et al. [8] explored the role of modern Hanfu in shaping tourist experiences, and iiMedia Research [9] highlighted the economic impact of modern Hanfu festivals, such as the Xitang Modern Hanfu Festival, which attracts millions of visitors annually.

Prior research [30,31] identified eight components of the experience for modern Hanfu tourism: social, cultural, historical, sensory, hospitality, functional, technological, and natural. This framework provides a comprehensive understanding of how modern Hanfu enhances the tourist experience.

#### Technological advancements.

Technological advancements have played a crucial role in the promotion and preservation of Hanfu culture. The development of virtual reality and augmented reality (AR) has provided immersive experiences for Hanfu. Reference [32] presented a Hanfu AR system that enables users to virtually try Hanfu using realistic cloth simulations. This technology enhances user experience and contributes to the preservation of traditional clothing. Moreover, in [32] the authors investigated the integration of digital twin technology with virtual try-on systems for traditional Chinese costumes and proposed a Kinect-based framework to address four critical challenges: fabric simulation, control, configuration, and calibration. This system demonstrates significant potential for applications in design, culture, education, and marketing. However, its functionality and precision in complex outfits and environments remain areas for improvement.

Additionally, a personalized recommendation system based on collaborative filtering algorithms can enable users to quickly identify content of interest. This reduces the time and energy spent on information organization by the platform, enhances users’ browsing efficiency and try-on experience, and further improves consumer experience on the marketing side [33].

These studies provide foundational insights for a “digital twin-based virtual try-on system” and a “modern Hanfu social platform user experience,” highlighting the need for interdisciplinary research and further optimization of system design.

#### Social media and identity.

Social media platforms have been instrumental in the spread of Hanfu culture, particularly among the younger generation. In [13] examined the discourse of Hanfu on platforms like Bilibili, highlighting its role in therapeutic governance and neo/non-liberal China. The use of Hanfu on social media is a way for individuals to express their cultural identity and connect with others who share similar interests.

Reference [33] explored the relationship between social media influence and the adoption of modern Hanfu, emphasizing the role of social media in shaping consumer behavior and cultural trends. The existing research on Hanfu has revealed a multifaceted approach to understanding traditional clothing. From its cultural significance and identity to its role in tourism and technological advancement, modern Hanfu continues to be a vibrant and evolving aspect of Chinese culture. The integration of modern Hanfu into various fields, including tourism, technology, and social media, highlights its enduring relevance and potential for future research.

Taken together, these three domains—cultural identity, technological advancement, and social media interaction—collectively construct the contextual foundation for modern Hanfu consumption. Cultural identity serves as the core symbolic driver, motivating consumers to express heritage and pride through clothing. Technological innovation, such as AR-based try-on and intelligent recommendation systems, provides both the tools and infrastructure for accessible and engaging participation. Meanwhile, social media functions as a mediating arena where cultural narratives are exchanged, aesthetic norms are shaped, and communities of practice are formed. These domains do not operate in isolation; rather, they dynamically interact. Social media platforms amplify cultural identity expression and enhance the reach and personalization of technology-based features. Technology, in turn, enables new modes of co-creation and consumer agency that reinforce both social belonging and cultural pride. This intersection of identity, media, and technology frames the rationale for our study: to explore how these factors jointly influence consumers’ online purchase intentions toward modern Hanfu, and how they may be theorized through integrated models such as TPB, SOR, and co-design frameworks.

### Theoretical model of relevant consumption behavior

Consumer behavior has been extensively studied using various theoretical frameworks, among which the theory of planned behavior (TPB) and the stimulus–organism–response (SOR) model are two of the most widely applied. TPB, proposed by Ajzen [34], posits that consumer behavior is driven by three key factors: attitudes toward the behavior, subjective norms, and perceived behavioral control. This model has been widely used to predict purchase intentions in various contexts, including sustainable fashion [35] and luxury goods [36]. However, TPB primarily focuses on individual psychological motivations and does not adequately account for the cultural and symbolic dimensions central to heritage-driven consumption, such as modern Hanfu. While TPB can explain the utilitarian aspects of purchase intention, it fails to capture the emotional and cultural identity factors that are critical in modern Hanfu, where consumers are often motivated by a desire to connect with traditional Chinese culture and express cultural pride [7,29].

Similarly, the SOR model, rooted in environmental psychology, suggests that external stimuli (e.g., marketing messages and product design) influence internal states (e.g., emotions and perceptions), which in turn drive behavioral responses (e.g., purchase intention). This model has been applied to online shopping environments to explain how website design and social media content influence consumer behavior [37,38]. While the SOR model provides a useful framework for understanding the impact of external stimuli on consumer behavior, it does not fully address the cultural and symbolic dimensions central to modern Hanfu consumption. Specifically, the SOR model tends to focus on immediate environmental stimuli and their emotional impact rather than the deeper cultural and identity-related motivations that drive modern Hanfu purchase decisions.

Given these limitations, this study adopts a more comprehensive approach by integrating cultural identity and co-design into the analysis of purchase intention. Although the TPB and SOR provide valuable insights into consumer behavior, they are not sufficient to fully explain the complex interplay between cultural, emotional, and utilitarian factors that influence modern Hanfu consumption. Therefore, this study builds on these models and extends them by incorporating additional dimensions, such as cultural identity and co-design, to better capture the unique motivations of modern Hanfu consumers. This approach allows for a more nuanced understanding of how cultural heritage and modern consumer behavior intersect in modern Hanfu.

### Online purchase intention

Purchase intention is a critical concept in consumer behavior research and represents the likelihood that a consumer will engage in a specific purchasing action. Given the vast number of studies on “online purchase intention,” this study employs a knowledge graph-based approach to analyze research hotspots and trends in this field. This approach enhances the data analysis efficiency by facilitating the identification of emerging research trends and hotspots in the field [39,40].

We retrieved 2,687 publications from the Web of Science (Core Collection) and the Emerald search system based on specific search criteria. The search conditions were as follows: (a) Title: “purchase intention” OR Title: “willingness to pay” AND Topic: “fashion online shopping” OR Topic: “textile e-commerce.” This study focuses on the literature related to consumer purchase intention in textiles, apparel, and fashion products.

#### Publication status.

**Fig 2:**
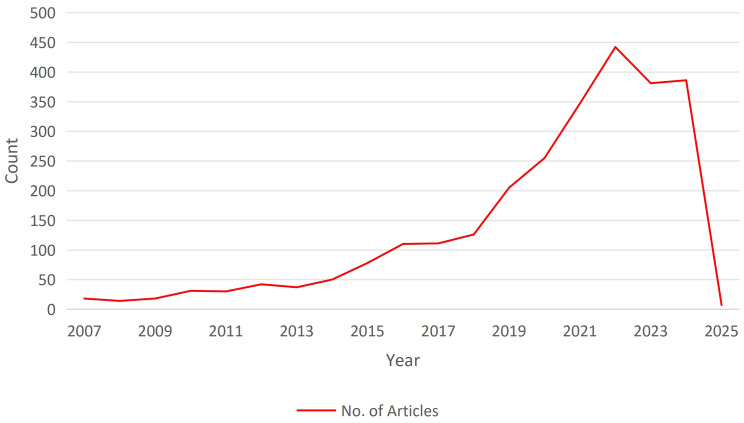
Publication trends in the field of online purchase intention research among fashion consumers.

Statistics show that the volume of publications in the field of online purchase intention among fashion consumers has grown significantly since 2016. From that point until 2022, the average annual growth rate reached 25.97%, as shown in Fig 2. However, a noticeable decline was observed in 2023. This trend reflects the substantial progress made in this research domain over the seven years from 2016 to 2022.

#### Core author analysis.

**Fig 3:**
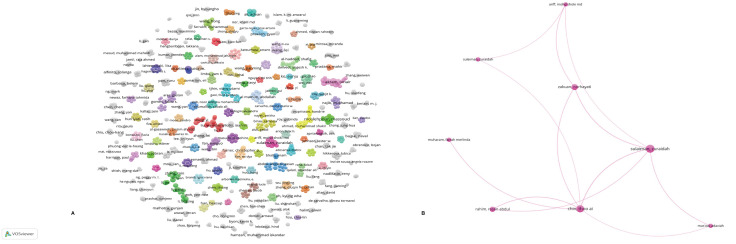
Core author analysis. (A) Author collaboration network. (B) Core author relationships.

By analyzing the core authors, we can identify the key research focus of purchase intention. Generally, the publications of core authors reflect the research directions within the field. By configuring the relevant parameters in VOSviewer, we generated a collaborative network of authors in this domain (Fig 3).

In the figure above, the size of the nodes represents the number of publications by each author, whereas the connections between nodes indicate collaboration among authors. Overall, research on online purchase intentions among fashion consumers exhibits relatively weak collaboration. There is a lack of extensive cooperation among core authors, and research is primarily conducted in small groups. In addition, many peripheral researchers have published only a limited number of studies. From a long-term perspective, academic exchange and collaboration are essential for advancing research on purchase intentions to new heights. According to the statistical analysis, the top 30 core authors (those with at least seven publications) collectively published 265 papers, accounting for 9.86% of the total 2,687 sample publications. The most prolific author is Zuraidah Sulaiman, who published nine papers between 2014 and 2024 focusing on topics such as electronic word-of-mouth, social media, green purchase intention, and sustainable apparel. The second most prolific author is Massoud Moslehpour, with seven publications between 2018 and 2021 that primarily researched social media marketing, perceived value, relationship marketing, and brand equity. The third-ranked author is Umair Akram, who published five papers from 2021 to 2024, mainly on social media, word-of-mouth, and green advertising. A comparative analysis of the research conducted by the core authors indicates that consumer-perceived value and social media are key research hotspots in this field.

#### Core institution analysis.

By analyzing core research institutions and their collaborative relationships in the field of online purchase intention research among fashion consumers, we can gain insights into the forefront of research in this domain. We generated an institutional collaboration network by setting relevant parameters in VOSviewer. Statistical analysis of this network provides information on the core institutions conducting research on online purchase intentions among fashion consumers worldwide from 2007 to 2025. Table 1 presents the top 29 institutions with the highest publication volume (≥ 15 papers).

**Table 1:** Core institutions in global online purchase intention research among fashion consumers (2007–2025).

Affiliations	Count	percentage
Universitas Bina Nusantara	40	1.49%
Indian Institute of Management Iim System	30	1.12%
Universiti Sains Malaysia	29	1.08%
Universiti Teknologi Mara	29	1.08%
University of Indonesia	29	1.08%
Universiti Teknologi Malaysia	25	0.93%
Hong Kong Polytechnic University	22	0.82%
Shenzhen University	22	0.82%
Wuhan University	21	0.78%
Multimedia University	20	0.74%
Shanghai Jiao Tong University	20	0.74%
Shanghai University	20	0.74%
Asia University Taiwan	19	0.71%
Chinese Academy of Sciences	19	0.71%
National Institute of Technology Nit System	19	0.71%
Sichuan University	19	0.71%
Universiti Putra Malaysia	19	0.71%
Shandong University	18	0.67%
Yonsei University	18	0.67%
California State University System	17	0.63%
Huazhong University of Science Technology	17	0.63%
National Kaohsiung University of Science Technology	17	0.63%
Jiangnan University	16	0.60%
Universiti Malaya	16	0.60%
Zhejiang University	16	0.60%
Indian Institute of Technology System Iit System	15	0.56%
Jilin University	15	0.56%
National Economics University Vietnam	15	0.56%
Symbiosis International University	15	0.56%

The top 29 core institutions collectively published 597 papers between 2007 and 2025, accounting for 22.22% of the total 2,687 sampled publications (Table 1). Among these, Universitas Bina Nusantara had the highest number of publications (n = 40), followed by the Indian Institute of Management (n = 30) and Universiti Sains Malaysia, Universiti Teknologi Mara, and the University of Indonesia (all n = 29).

The geographic concentration of research institutions in Southeast Asia—particularly in Indonesia and Malaysia—reflects a strong regional interest in digital fashion commerce and online consumer behavior. However, this also reveals a potential limitation in the generalizability of existing findings, especially when applied to culturally symbolic products like modern Hanfu, which are deeply rooted in Chinese traditions. The underrepresentation of Chinese institutions and limited cross-national participation in the top-contributing entities may weaken the contextual grounding of theoretical models in this specific cultural domain. In addition, the collaboration network analysis showed relatively sparse connections among core authors, highlighting a lack of deep interdisciplinary and inter-institutional collaboration. These issues are important because strong geographic diversity and academic collaboration are essential for enhancing theoretical innovation, cultural adaptability, and empirical robustness in the study of symbolic fashion consumption.

#### Keyword co-occurrence analysis.

Keywords are the precise extraction of the essence of a paper, and studying keywords can accurately identify research hotspots and evolving trends in the current research field. Using VOSviewer to analyze the titles and abstracts of the sampled literature, we obtained clustering information on keywords in the field of online purchase intention research among fashion consumers. VOSviewer clustered keywords into five clusters. We selected two keywords from each cluster based on the weight of occurrence, ordered from highest to lowest (Table 2).

**Table 2:** Keyword clusters.

Label	Cluster	Weight (Occurrences)
Attitude	1	628
User	2	291
Use	5	270
Originality value	4	250
Knowledge	1	244
Design methodology approach	4	228
Type	2	224
Technology	3	189
System	3	133
Usefulness	5	128

**Fig 4:**
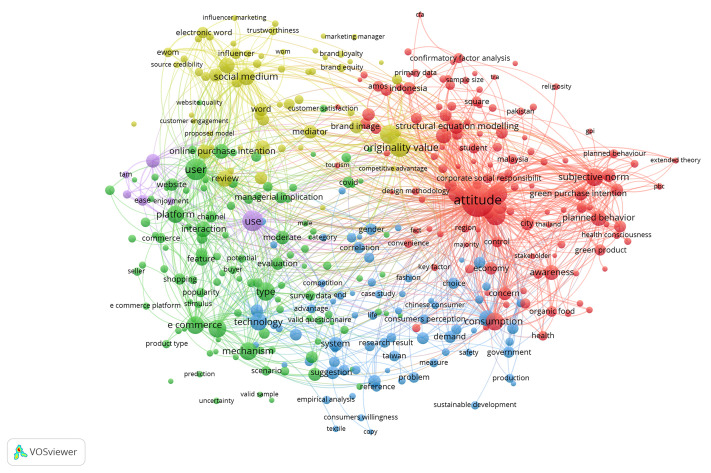
Keyword co-occurrence network.

The co-occurrence network obtained through keyword co-occurrence analysis is shown in Fig 4.

Different colors represent different clusters. From the color distinction, we observed that there were approximately five clusters, indicating that the sampled literature contained five categories of keyword clusters. The sizes of the circles represent the frequency of keyword occurrence; larger circles indicate a higher frequency of occurrence of the corresponding keywords.

The clusters revealed by the keyword co-occurrence analysis align closely with core constructs in theoretical models such as TPB and SOR. As shown in Fig 4, terms like “attitude,” “subjective norm,” and “planned behavior” reflect TPB constructs, while keywords such as “interaction,” “emotion,” and “platform” mirror the Stimulus–Organism–Response structure. These findings support the integration of emotional, functional, and social dimensions into our proposed model of Hanfu purchase intention.

#### Key nodes in the co-occurrence network.

**Fig 5:**
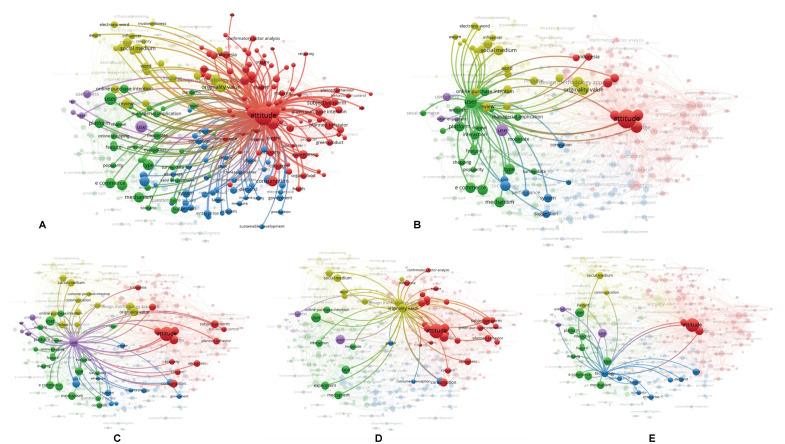
Key nodes in the keyword co-occurrence network. (A) The co-occurrence network with “attitude” as the key node. (B) The co-occurrence network with “user” as the key node. (C) The co-occurrence network with “use” as the key node. (D) The co-occurrence network with “originality value” as the key node. (E) The co-occurrence network with “technology” as the key node.

The first keyword cluster revolves around attitude, with related terms such as consumption, subjective norms, awareness, green purchase intention, and planned behavior, highlighting several key research points related to attitude (Fig 5). Similarly, the second keyword cluster focused on users with terms such as e-commerce, mechanism, platform, type, and interaction. The third cluster centers on use, with associated terms such as ease, usefulness, technology acceptance model, subjective norm, and consumption. The fourth cluster focuses on originality value with keywords such as design methodology approach, social media, word-of-mouth, mediator, and communication. Finally, the fifth cluster revolves around technology, with terms such as performance, system, platform, demand, and enterprises.

Fig 5 illustrates five distinct keyword clusters, each representing different aspects of consumer purchase research. Cluster 1 reflects cognitive drivers (e.g., attitude, awareness); Cluster 2 emphasizes platform-based interaction; Cluster 3 focuses on technology acceptance; Cluster 4 highlights communication-based influence; and Cluster 5 centers on performance and system demand. These thematic areas correspond to the psychological and contextual layers modeled in TPB and SOR frameworks. This interpretation moves beyond descriptive statistics to demonstrate theoretical alignment.

#### Dynamic evolution of keywords.

**Fig 6:**
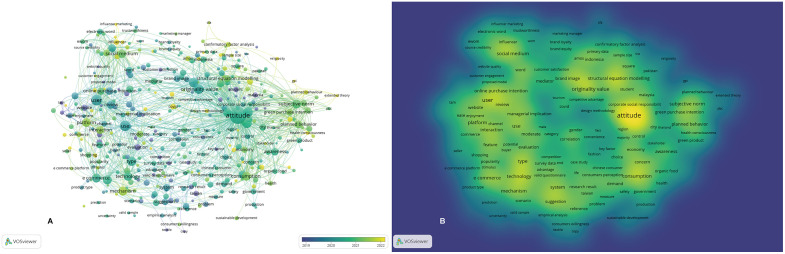
Keywords dynamic evolution. (A) Keywords overlay visualization. (B)Keywords density visualization.

To observe the evolution of keywords over time visually, VOSviewer was used to generate a keyword timeline for the sampled literature (Fig 6A). The node colors represent research hotspots at different stages, with each node’s line indicating the flow of knowledge over time, transitioning from blue-purple to yellow. In Fig 6A, the blue-purple nodes represent research conducted before 2019, whereas the yellow nodes represent research conducted after 2022. The more a keyword node leans toward yellow, the more it indicates that the research field is moving toward the forefront. By observing the color change, it is evident that the field has shifted from more generalized research areas, such as attitude, user, originality value, and technology, to more specific topics, including health consciousness, mechanism, COVID, influencer, interaction, and platform. These newer keywords suggest a growing academic focus on technology-mediated social influence, user-platform dynamics, and context-sensitive risk perception in fashion consumption. This temporal evolution supports the rationale for incorporating more dynamic and interaction-based constructs (e.g., social media engagement, co-design) into contemporary purchase intention models, particularly for symbolic or culturally driven products like modern Hanfu.

Additionally, using density visualization (Fig 6B) to observe the current research density, yellow represents high-density areas, indicating regions with a high concentration of literature, whereas blue represents low-density areas. It is clear from the visualization that keywords such as attitude, social media, user, consumption, and e-commerce are in the high-density areas. This indicates that the core research focus in fashion purchase intention literature remains centered around individual psychology, platform usage behavior, and digitally mediated consumption. The central placement of “social media” and “user” suggests that consumers are increasingly viewed not only as passive recipients of marketing stimuli but as active participants in meaning-making processes, aligning with the SOR model’s evolving application in interactive and participatory media environments. Additionally, the dense clustering of “attitude” and “subjective norm” continues to affirm TPB’s foundational role in framing consumer behavior, yet the emergence of “co-creation,” “engagement,” and “influencer” (albeit at lower density) signals a methodological and theoretical shift toward recognizing socially constructed value and co-designed experiences.

#### Main research perspectives.

**Fig 7:**
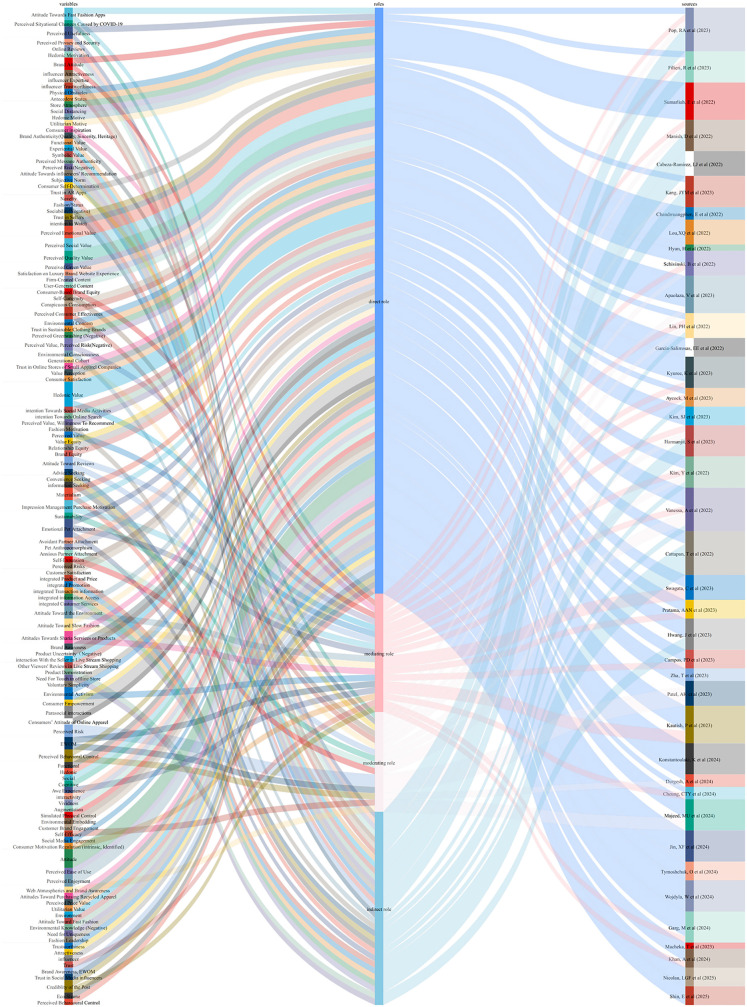
Main research perspectives of the sampled literature.

This study selected 39 papers published between 2022 and 2025 from the sampled literature for an in-depth analysis (Fig 7). The Sankey figure visually represents the categorization and distribution of variables related to “online purchase intention” across the selected studies. On the left side of the figure, key variables identified in the literature are depicted, including prominent constructs such as attitude, perceived social value, perceived quality value, hedonic value, and perceived risk, among others. The middle section of the figure illustrates the roles these variables assume within their respective studies, categorized into four types: direct role, mediating role, moderating role, and indirect role. Notably, variables assigned a direct role dominate the distribution, accounting for approximately 60% of all role types. This indicates that these variables exert the most immediate and significant influence on “online purchase intention.” The right side of the figure traces the origins of these variables and roles to the respective studies, providing a clear mapping of their scholarly sources. This visualization offers a comprehensive overview of how variables and their roles are distributed across the literature, highlighting patterns and trends in the conceptualization of “online purchase intention.”

Several factors influence purchase intention across various contexts. Economic factors such as perceived value and perceived risk play significant roles. For instance, perceived value, which encompasses functional, emotional, social, and environmental dimensions, positively influences purchase intention in studies of second-hand luxury goods and recycled apparel [41,42]. Similarly, perceived risks, including functional, aesthetic, and sanitary risks, negatively affect purchase intentions in second-hand clothing [43].

Social and cultural factors also play crucial roles. Generational cohort theory suggests that different generations have varying levels of environmental consciousness, which influence their purchase intentions toward sustainable fashion [44]. Additionally, the COVID-19 pandemic has accelerated the shift toward online shopping, with omni-channel integration significantly influencing consumer satisfaction and purchase intentions in the fashion industry [45].

Purchase intention is often examined through various dimensions including utilitarian, hedonic, and social values. Utilitarian values refers to the practical benefits that consumers derive from a product, such as efficiency, convenience, and cost-effectiveness [46]. In the context of Hanfu, this may include wearability, ease of maintenance, and compatibility with daily life in urban settings.

Hedonic values captures the emotional and experiential pleasure derived from consumption, particularly in the fashion industry [47,48]. For modern Hanfu, hedonic value is often associated with aesthetic enjoyment, participation in cultural festivals, and feelings of nostalgia or pride linked to traditional Chinese attire.

Social value reflects the extent to which wearing Hanfu enhances one’s social image or helps gain recognition from peers. It is particularly relevant in online communities where users share Hanfu styling and participate in cultural discourse [49–51].

Perceived risk denotes the potential negative outcomes associated with a purchase [52]. For Hanfu consumers, this may include sizing inaccuracy, fabric inauthenticity, or social misunderstanding (e.g., fear of cultural misrepresentation or being misinterpreted in public settings).

These dimensions are critical for understanding symbolic consumption, where the value of the product extends beyond utility and is deeply embedded in identity expression, cultural continuity, and emotional resonance [53]. In the case of modern Hanfu, symbolic consumption manifests through practices that intertwine historical aesthetics with personal and collective identity. Consumers may choose Hanfu not solely for functional use but as a means of expressing cultural pride, participating in rituals (e.g., festivals, ceremonies), or connecting with like-minded subcultural communities online. The symbolic value of Hanfu therefore resides in both its material attributes (e.g., silhouettes, motifs) and the sociocultural meanings consumers attach to it.

Moreover, these dimensions are intricately linked to constructs such as cultural identity and co-design. For example, hedonic value contributes to cultural identity by enabling emotional engagement with traditional aesthetics, fostering a sense of belonging and pride in one’s heritage. Social value reinforces identity formation by facilitating peer recognition and community validation, particularly in digital spaces where Hanfu enthusiasts exchange styling ideas and cultural narratives. Utilitarian value, though often perceived as functional, also supports identity when consumers integrate Hanfu into daily life—normalizing cultural expression in contemporary settings. Perceived risk, conversely, can inhibit identity expression when consumers fear cultural misinterpretation or judgment.

These variables also interact with co-design dynamics. Co-design practices, which invite consumers to participate in product customization or collaborative creation, heighten hedonic and social value by increasing emotional attachment and social involvement. At the same time, co-design can mitigate perceived risk by offering better fit, clearer communication, or culturally respectful designs. Thus, the relationship between these variables and cultural identity/co-design is not only conceptually coherent but also practically observable in Hanfu consumption behaviors.

In light of the above discussion, the literature review revealed that factors such as social media engagement, product quality, and cultural identity play significant roles in shaping consumers’ purchase intentions toward modern Hanfu. While the existing research has extensively explored the psychological motivations behind consumer behavior, the influence of brand communication, and the impact of social media on purchasing decisions, a gap remains in the comprehensive analysis of these factors within specific cultural contexts. Particularly in the case of modern Hanfu, despite some research on traditional cultural products, there has been limited exploration of how to integrate online consumer reviews, sentiment analysis, and quantitative methods.

This study seeks to fill this gap by employing a combined approach of text and quantitative analyses to extract key factors from consumer reviews and develop a purchase intention model. This methodology offers a more nuanced understanding of cultural consumption and provides practical insights for modern Hanfu brands to formulate effective marketing strategies.

## Materials and methods

This study employed a sequential mixed-methods design, integrating qualitative and quantitative approaches to explore the drivers of modern Hanfu purchase intention in digital commerce. The research comprised three interconnected studies: Study 1 utilized text mining to analyze a large corpus of online consumer reviews, identifying initial themes and salient factors. Study 2 applied grounded theory to the same textual data to inductively develop a theoretical model and conceptual dimensions through systematic coding. Building on the insights from Studies 1 and 2, Study 3 involved the development and validation of a measurement scale, followed by a quantitative survey to test the structural relationships between the identified factors and purchase intention using structural equation modeling (SEM). This design allowed for a data-driven exploration of factors followed by empirical validation.

**Fig 8:**
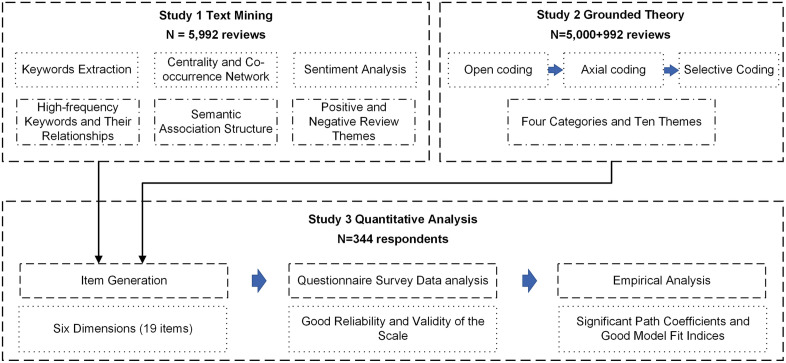
Sequential mixed-methods framework.

To ensure a rigorous and data-driven exploration, the three studies are sequentially designed and tightly integrated, forming a methodological triangulation that leverages the strengths of each approach, as shown in Fig 8.

### Study 1 text mining

#### Consumer review collection and purification.

Because of the characteristics of modern Hanfu’s online review data, such as a narrow closed surface, high concentration, and uniform format, the application of text-mining technology to analyze online reviews of modern Hanfu consumers can comprehensively, deeply, and accurately understand the features of the target market and the demands of consumers [54]. This study employs text-mining technology based on the Chinese text natural language processing library (Python SnowNLP) to analyze online reviews of modern Hanfu consumers. Text mining is a research technique based on natural language processing that plays a crucial role in the collection, processing, and analysis of textual data. It provides deep insights into consumer experiences and needs [55,56].

This study selected nine modern Hanfu brands, each with sales exceeding 500 units and more than 200 reviews on JD (https://www.jd.com) and Taobao (https://www.taobao.com) e-commerce platforms. Data collection and processing for each brand were performed in four steps. The first step involved using data collection software to gather consumer review data from January to December 2023 for the aforementioned nine modern Hanfu brands, as listed in Table 3. The second step was to remove 86 duplicate and invalid entries (e.g., “standard positive reviews”). The third step involves deleting special emojis and symbols from the data. The fourth step was to convert the data from traditional to simplified Chinese. Consequently, 5,992 valid reviews were obtained.

**Table 3:** Data information description.

Brand	Code	Product quantity	Number Of Reviews	Percentage%
AMW	A	2	550	9.17
Suyuhuashang	B	3	644	10.74
Baohongsi	C	2	1225	20.44
Geshidun	D	2	740	12.35
Rindu	E	2	785	13.10
VKGZ	F	1	381	6.36
Dingmaodiao	G	2	564	9.41
Kaji	H	1	259	4.32
Zhenyimeng	I	2	844	14.10

#### Keywords extraction and cross-analysis.

This study used the KH Coder 3 text analysis software to extract keywords in four steps. First, the data were imported, and meaningless words, such as “I,” were filtered out. Second, “mandatory” extracted words were added to the dictionary to prevent colloquial reviews from being split into individual words during the tokenization process, ensuring the comprehensiveness of the keyword extraction. Third, keywords, including nouns, adjectives, and related words, were extracted and their word frequencies were calculated. If there was repeated content (e.g., “good good good”), it was counted only once. Finally, since the meanings of words may vary in different contexts or usages, the meanings of the keywords were verified. This verification process used a software access function to confirm whether the meanings of the keywords aligned with the original text. Manual corrections were performed if discrepancies were observed.

Considering the data volume and structure, this study extracted 30 high-frequency keywords with a word frequency exceeding 140 and practical significance [56]. Subsequently, two faculty members from a university fashion department discussed and classified keywords based on their intrinsic characteristics.

To examine the distribution and prominence of high-frequency keywords across different brands, a cross-analysis was performed using KH Coder 3. This analysis visualized the association between the nine brands (A-I) and the keywords, generating a matrix that indicated the relative frequency and distribution of each keyword within each brand’s reviews.

#### Centrality analysis.

Centrality is a core concept that is used to measure the importance or influence of a node in a network. Centrality quantifies a node’s central position in a network using various indicators [57]. This study uses two centrality measures—degree centrality and betweenness centrality—to identify the most important and influential keywords in the collected consumer review data.

Degree centrality is a social network analysis method that measures the centrality of a node by evaluating its direct connections and reflecting its influence or activity level within the network [57]. Betweenness centrality is an indicator used to measure the intermediary or bridging roles of the nodes in a network. This reflects the node’s ability to control the flow of information and resources [57,58].

#### Co-occurrence network analysis.

Co-occurrence analysis focuses on the frequency of multiple words appearing together in a text, thereby revealing the relationships between words, theme similarities, and semantic correlations. This approach uncovers the structure of the data and discovers its themes and underlying meanings [56]. To elucidate the primary themes and their intrinsic characteristics, this study applies a co-occurrence analysis to the collected consumer review data and visualizes the results.

#### Sentiment analysis of consumer reviews.

Although the aforementioned text-mining methodologies—including keyword frequency analysis, cross-network mapping, centrality evaluations, and co-occurrence network assessments—provide structural insights into textual patterns, they fall short of discerning the sentiment polarity inherent in consumer evaluations. Empirical studies have established that purchasing decisions are asymmetrically influenced by evaluative language, with negative reviews exerting disproportionately stronger effects than their positive counterparts [59–61]. To address this limitation, we employed Python’s SnowNLP toolkit, which is a probabilistic natural language processing library optimized for Chinese sentiment analysis. SnowNLP assigns a sentiment score between 0 and 1 to each review, with scores above 0.5 classified as positive and scores below 0.5 as negative, based on its pre-trained Bayesian model. This allowed for the categorization of all reviews into positive and negative sentiment classes for further analysis. By systematically categorizing modern Hanfu reviews into binary sentiment classes, this analytical framework prioritizes the granular examination of negative evaluations—a critical yet underexplored dimension in the existing text-mining literature—thereby uncovering actionable insights obscured by purely relational or frequency-based approaches.

Subsequent to this classification, two in-depth textual analyses were performed on the separated positive and negative reviews:

(1) Keyword Frequency Analysis for Positive and Negative Reviews: The KH Coder 3 software was used again to extract nouns, adjectives, and verbs from the positive and negative review datasets separately. The frequency of occurrence for each keyword within each sentiment category was then calculated and ranked.
(2) Co-occurrence Network Analysis for Positive and Negative Reviews: Separate co-occurrence network analyses were conducted on the high-frequency keywords identified in the positive and negative reviews. This aimed to uncover the thematic structures and semantic associations unique to consumers’ satisfactory and critical feedback, respectively. The networks were visualized to illustrate the distinct patterns of word linkages in each sentiment group.

### Study 2 grounded theory

Although the above analysis based on text mining can extract structured information from a large amount of text data to reveal the deep laws of consumer behavior, attitudes, and needs, the above analysis relies on the semantics of the review text, and its core research object is the literal meaning of linguistic symbols, which belongs to the category of semantics, which is concerned with “the meaning of language itself,” which is detached from language use [62,63]. In the modern Hanfu consumption scenario, consumer reviews reflect consumers’ core demands and basic value judgments about the product; however, these demands and judgments do not exist in isolation but are deeply embedded in the specific context of language use. For example, “quality” and “like,” “good-looking,” “fabric,” and other words appear frequently, and cross-tabulation analysis shows that “quality” was evenly distributed and accounted for the largest proportion of the nine brands, while “fabric” led the network analysis in degree centrality and betweenness centrality, along with “comfort” “size” and “fit” “size” and “production process” form a strong correlation. However, the results of sentiment analysis show that in 6.3% of the negative reviews, words such as “quality,” “fabric,” and “production techniques” appear centrally, suggesting that there are hidden risks behind the superficial praise. However, these explicit data cannot explain deeper motives.

Therefore, it is essential to further contextualize language use within its dynamic environment; modes of consumer expression, interactional contexts, and cultural symbols collectively form fertile ground for meaning creation. To transcend the limitations of static semantic analysis, this study introduces grounded theory, employing a three-tiered coding process—open coding, axial coding, and selective coding—to inductively derive, from review data, the framework of consumers’ value recognition of modern Hanfu (e.g., “cultural heritage” or “everyday practicality”), the logic of emotional drive (e.g., “national pride” or “in-group affiliation”), and conflicts in decision-making (e.g., balancing price sensitivity with quality pursuit). Only by recontextualizing linguistic symbols within their original sociocultural milieu can we decipher the complex landscape of consumer intentions underlying modern Hanfu consumption.

Grounded theory advocates bottom-up theory generation from raw data, avoiding the interference of preconceived frameworks, and constructing realistic theoretical models from fragmented empirical data. The strength of this methodology makes it an ideal tool for exploring emerging cultural consumption phenomena. Not only can it analyze the logic of meaning, dynamic interactions, and implicit social relationships implied in the phenomena, but it can also penetrate the surface text to explore the underlying motivations (e.g., affective symbols, hierarchical identity metaphors, etc.) that are not directly stated in the descriptions of consumers’ purchasing experience [64,65]. To address the special characteristics of modern Hanfu consumption research: dynamism: market trends are rapidly iterating, and it is difficult for preconceived theories to cover emerging needs; context-dependence: consumers’ interpretation of high-frequency words such as “quality” and “good-looking” is highly dependent on the cultural rules of modern Hanfu communities; and implicit contradiction: the text mining reveals that “high positive emotions coexist with negative feedback on quality,” but it cannot explain its conflict mechanism. Therefore, this study adopts the grounded theory to encode the cleaned review data at three levels, aiming to break through the limitations of the traditional discourse analysis of text mining, building a theoretical framework rooted in local consumption scenarios, and reveal the deep decision-making logic from “symbolic preference” (e.g., intensive distribution of “good-looking” of brand B) to “quality anxiety” (e.g., negative feedback on “threads” “production techniques” etc.).

**Fig 9:**
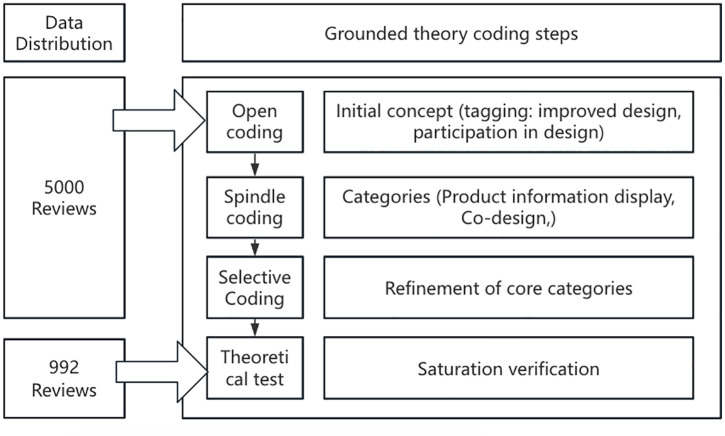
Grounded theory analysis path.

Based on the grounded theory methodology, this study implements a systematic coding analysis of the cleaned consumer review data, which contains a two-phase research design of exploratory and validation analyses (Fig 9).

The specific operational procedures were as follows. Phase 1: exploratory coding analysis (n = 5000). Open coding: A coding team comprising two university teachers specializing in clothing science was formed to analyze comment texts individually using consensus coding and to extract explicit/implicit elements through semantic unit slicing and concept labeling. To enhance the reliability and trustworthiness of the coding process, we applied a multi-step validation approach. The two coders independently coded the same set of samples, and intercoder reliability was assessed using percentage agreement across categories, which exceeded 85%. Discrepancies were discussed and resolved through consensus meetings moderated by a third senior researcher with experience in qualitative research methodology. To mitigate bias, both coders received training in grounded theory techniques prior to the study and were instructed to bracket personal assumptions to ensure that concepts emerged inductively from the data rather than preconceived frameworks. For example, in response to the comment “Last year’s sleeves were too big, this year’s big sleeves have the feeling of ancient clothes and are easy to move, very good, primarily featuring listening to advice,” the initial concepts such as “corporate responsiveness (listen to advice),” “product iteration (improvement)” and “user participation (adoption)” are extracted. Axis coding: Clustering categories based on logical associations between concepts to form a thematic framework. A typical example is the integration of “listening,” “improvement” and “adoption” into the theme of “consumer co-design,” which reveals the driving mechanism of user participation in product optimization. Selective coding: A theoretical saturation test is used to screen the core categories, eliminate the dimensions of “cultural identity” and “product quality” that are duplicated in the previous text mining, and finally constructs four innovative theoretical dimensions, such as “product information display” and “co-design,” to form a preliminary theoretical framework. Phase 2: Validation of the coding analysis (n = 992). Back-to-back coding was conducted on independent subsamples and no new concepts or categories were detected, indicating that the framework achieved theoretical saturation [65].

### Study 3 quantitative analysis

#### Item generation.

Building on textual insights from Study 1 (n = 5,992 reviews), which identified fragmented keywords through frequency analysis and sentiment mining, Study 2 conducted a parallel grounded theory analysis on the same dataset to establish structured dimensions. While Study 1 revealed atomic-level descriptors like “quality” “fabric” and “logistics” through computational linguistics, Study 2 systematically applied three-level coding procedures (open-axial-selective) to develop higher-order conceptual categories. This dual analytical approach, combining data-driven text mining with theory-building grounded analysis, yielded complementary perspectives. Study 1 provided granular lexical patterns, whereas Study 2 synthesized these patterns into four coherent dimensions through rigorous semantic interpretation. The resultant framework, which integrates the empirical specificity of text mining with the theoretical depth of grounded analysis, establishes a multidimensional foundation for the quantitative validation of Study 3.

To further refine and quantify these insights, Study 3 employed a more targeted approach, organizing the extracted themes into specific dimensions. Drawing from text analysis, text coding, and existing literature on Hanfu and purchase intention, we identified nine key factors that encapsulate the underlying motivations driving consumers’ decisions in modern Hanfu. These dimensions were systematically categorized through expert evaluations and focus group discussions. The nine dimensions provide a comprehensive framework that reflects the various aspects of consumer behavior and informs construction of a detailed measurement scale, which will be tested in the subsequent quantitative analysis.

While TPB and SOR were introduced earlier to contextualize the broader theoretical landscape, we intentionally departed from directly applying their specific constructs (e.g., perceived behavioral control, organismal response states) in Study 2 and Study 3 for two key reasons. First, TPB and SOR key variables such as “hedonic value” and “perceived risk” were not dominant in our grounded theory results. Second, modern Hanfu is a culturally symbolic fashion category, where consumer behavior is shaped by community aesthetics, participatory design, and symbolic value construction—dimensions not well represented by TPB or SOR’s original psychological structure. Instead, we retained the structural logic of these models—namely, that external and internal stimuli jointly shape behavioral intention—as a conceptual scaffold. Therefore, the final measurement dimensions reflect the Hanfu-specific consumer landscape while remaining compatible with broader behavioral models in structure.

This study organized a focus group comprising doctoral professionals in fashion design and brand marketing (n = 5) and industry practitioners (n = 2). Forty-four statements were developed based on nine dimensions, and their accuracy and necessity were discussed. Subsequently, the 44 statements were shuffled and sent to three senior researchers for further analysis. The researchers were asked to a) consider the accuracy and necessity of these 44 statements and check for any ambiguity in their expressions, and b) categorize these 44 statements into nine dimensions. If at least two researchers placed a statement on the same dimension, the statement was retained. After the process was completed, 25 statements were deleted and 19 statements were retained.

#### Questionnaire collection and sample analysis.

An online questionnaire was distributed to consumers who had purchased modern Hanfu in the past year using an online survey platform. The measurement items used a seven-point Likert scale, where 1–7 represent responses ranging from “strongly disagree” to “strongly agree.” Before starting the questionnaire, participants were informed that the data collected would be used solely for academic research purposes, and submission of the questionnaire indicated their consent to this notice. A cash reward was offered upon the completion of the questionnaire. Data collection was conducted between September 13, 2024, and October 5, 2024. A total of 344 valid responses were obtained.

#### Reliability and validity analysis.

SPSS software (version 22.0) was used to test the reliability and validity of the scale. To assess the scale’s overall psychometric properties, we conducted preliminary tests for sampling adequacy and internal consistency. We assessed internal consistency using Cronbach’s alpha. The Kaiser–Meyer–Olkin (KMO) measure and Bartlett’s test of sphericity were used to confirm the suitability of the data for factor analysis. Convergent validity was evaluated using Average Variance Extracted (AVE), scale reliability was examined using composite reliability (CR), and discriminant validity was evaluated by comparing the square root of the AVE for each construct with its correlations with other constructs. Multicollinearity was checked using the Variance Inflation Factor (VIF).

#### Empirical analysis.

To further test the stability of the evaluation scale for consumers’ online purchase intention of modern Hanfu, this study used the SEM analysis software AMOS 24.0 for model fitting. Maximum likelihood estimation was used to estimate the path coefficients. Model fit was assessed using multiple indices: χ²/df, Root Mean Square Error of Approximation (RMSEA), Goodness-of-Fit Index (GFI), Incremental Fit Index (IFI), Tucker-Lewis Index (TLI), Normed Fit Index (NFI), and Comparative Fit Index (CFI).

## Results

### Text mining

#### Keyword frequency and thematic distribution.

The keyword “quality” has the highest word frequency, indicating its significant role in the dataset, followed by “like,” “good-looking,” and “fabric” (Table 4). The high frequency of keywords related to product quality suggests that product quality is a fundamental element in consumer evaluations of modern Hanfu products. By contrast, price-related keywords appeared much less frequently. This shift is attributed to changes in the mindset of Chinese consumers, who now place more emphasis on product quality and are willing to spend higher prices on superior products rather than blindly pursuing the lowest prices [66].

**Table 4:** The 30 highest frequency keywords.

Classes	Code	Key Words	Frequency
product quality(A)	A1	Quality	1604
	A2	Fabric	878
	A3	Comfortable	869
	A4	production techniques	827
	A5	Size	466
	A6	Packaging	157
	A7	Thread	147
	A8	Details	147
	A9	Chroma	146
	A10	Porosity	146
Product design (B)	B1	Like	1267
	B2	Good-looking	1169
	B3	Style	527
	B4	Complexion	504
	B5	Color	389
	B6	Shape	244
	B7	Design	183
Delivery efficiency (C)	C1	Fast	465
	C2	Logistics	330
	C3	Delivery	225
	C4	Speed	265
	C5	Publish	144
Merchant Services (D)	D1	Service	289
	D2	Attitude	293
	D3	Seller	193
	D4	Clerk	168
	D5	Photo	148
Price (E)	E1	Price	403
	E2	Deserve	280
	E3	Cost-effective	191
	E4	Affordable	155

#### Cross-analysis outcomes.

**Fig 10:**
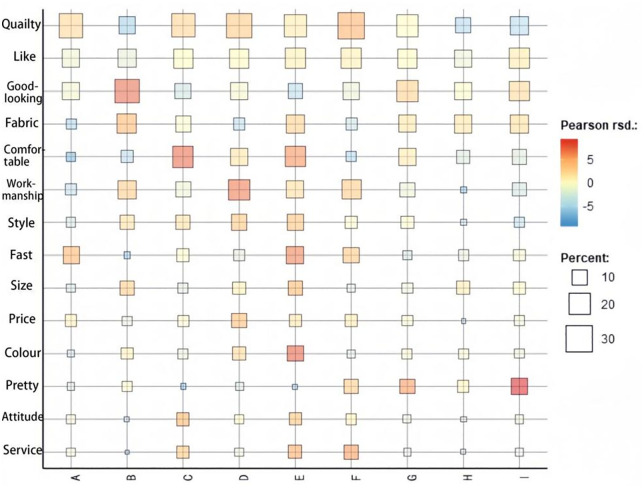
Keyword cross-analysis.

Fig 10 illustrates the results of the cross-analysis of the 14 high-frequency keywords from brands A-I. In the keyword cross-analysis chart, the squares represent the scale of the keyword coding applied, whereas the shading of the squares indicates the size of the Pearson’s relative standard deviation value. The darker the color, the more significant the deviation. From the figure, it is evident that “quality” occupies a large proportion across all nine brands, with the color generally being darker, indicating that the proportion of “quality” is substantial and its distribution is relatively even. This suggests that, for most brands, quality is the primary factor that consumers focus on. Next, “like” is evenly distributed and occupies a significant proportion of all brands, making it the second most important factor after “quality.” Third, “good-looking” also follows this pattern, especially in Brand B, where it has a large proportion and a very concentrated distribution, making it a key factor that consumers care about most.

#### Centrality and co-occurrence network.

**Fig 11:**
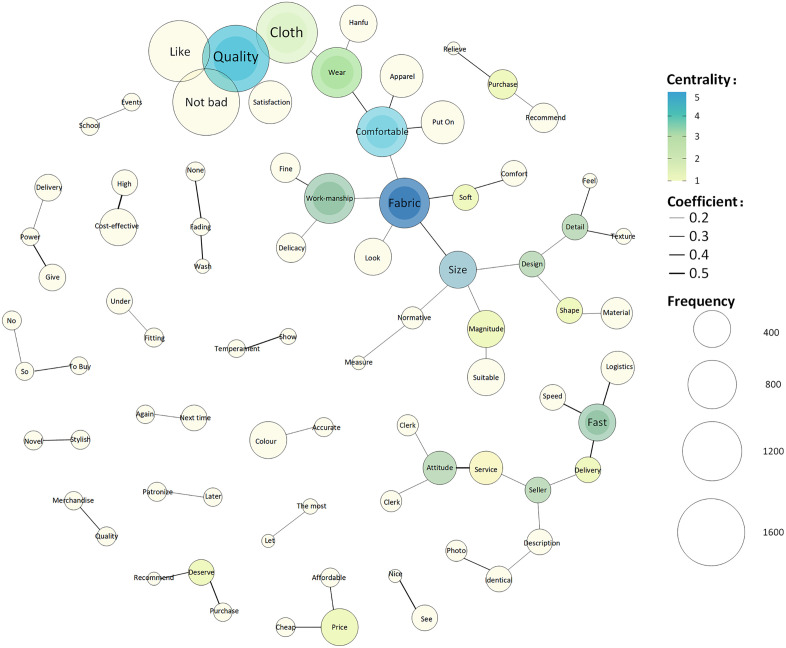
Degree centrality analysis.

**Fig 12:**
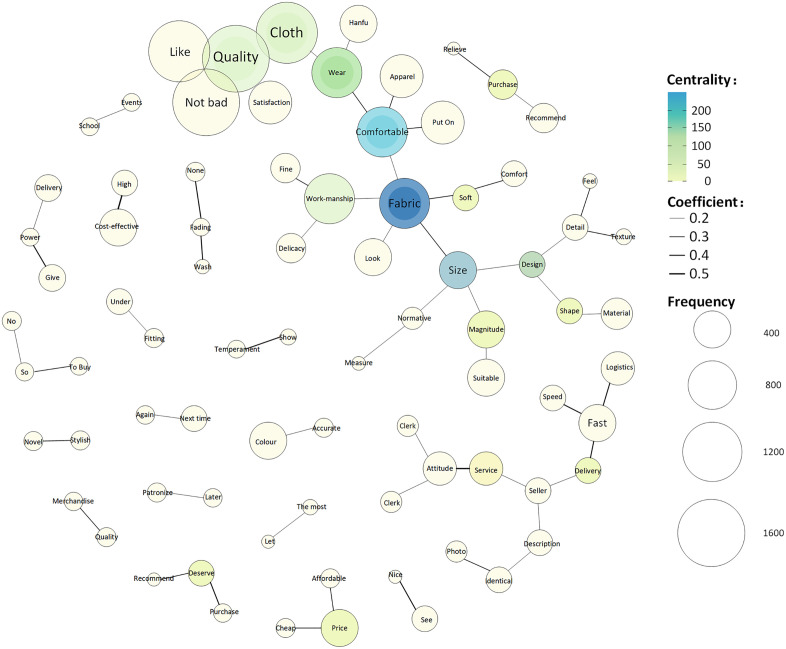
Betweenness centrality analysis.

In Fig 11, the size and color depth of the circles represent the frequency of keyword occurrences and centrality, respectively. The larger the circle, the higher is the frequency of occurrence, and the deeper the color, the higher is the centrality. The results show that the node for “fabric” has the most connections and the deepest color, indicating the highest centrality and greatest influence, followed by “comfort” “production techniques” “size” and “quality.” The keyword “fabric” has the deepest color, indicating the highest centrality, followed by “comfort” and “size” (Fig 12). Keywords with high centrality significantly influenced the direction of reviews. Therefore, “fabric,” “comfort,” and “size” are important considerations for modern Hanfu consumers during their purchasing process.

As noted above, “fabric,” “comfort,” and “size” show high degree centrality and betweenness centrality, indicating that they are the most influential and important keywords in the network.

**Fig 13:**
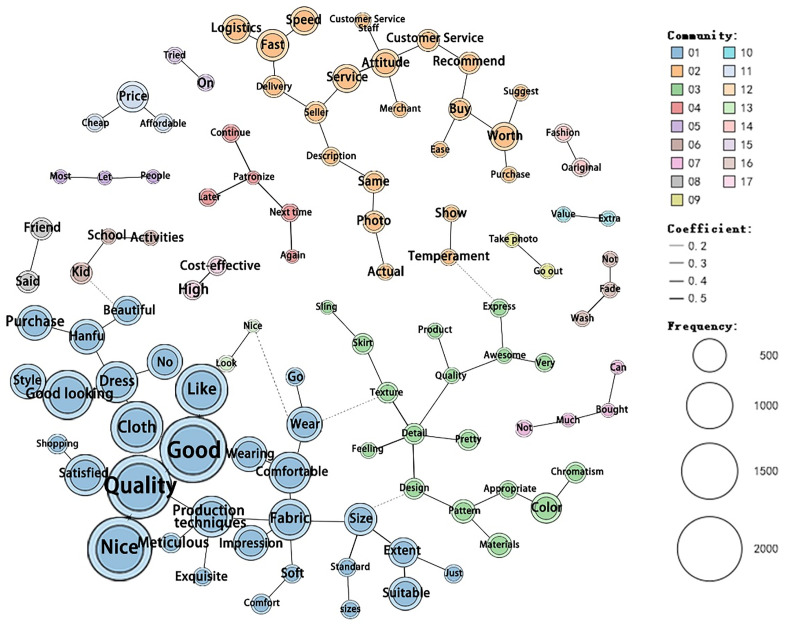
Keyword co-occurrence network.

In Fig 13, the size of the circles represents frequency, different colors indicate different themes, the solid line reflects the degree of correlation, and dashed lines represent the co-occurrence of keywords across different themes. For example, “quality” has direct connections with “good,” “beautiful,” “fabric,” and “satisfaction” indicating their high frequency and strong relationships. Therefore, consumer evaluations of modern Hanfu mainly include product quality (production techniques, fabric, and size), design (good-looking, style, and beautiful), price (affordable and cheap), delivery efficiency (logistics, fast, and speed), and customer service (attitude, service, and customer service staff).

#### Sentiment analysis and subsequent textual analysis outcomes.

Sentiment analysis classified 5,614 reviews as positive (93.7%) and 378 reviews as negative (6.3%) (Table 5), indicating that most consumers were satisfied with their purchases of modern Hanfu products.

**Table 5:** Brand satisfaction rates.

Brand code	A	B	C	D	E	F	G	H	I
**Positive feedback**	461	598	1149	703	758	381	517	241	806
**Total**	550	644	1225	740	785	381	564	259	844
**Satisfaction rate (%)**	83.8	92.9	93.8	95.0	96.6	100	91.7	93.1	95.5
R = 16.2%; σ = 4.4									

Note(s) R: The ratio of positive to negative reviews. σ: The standard deviation.

Table 6 lists the top 10 keywords for both positive and negative reviews. Notably, in the positive reviews, keywords related to product quality had the highest cumulative frequency, appearing 4,559 times, followed by those related to product design, which appeared 4,464 times. Therefore, the primary factor driving consumers’ positive evaluation of modern Hanfu products is product quality, followed by product design.

**Table 6:** Keyword frequency in positive and negative reviews.

Positive comments		Negative comments	
Words	Frequency	Words	Frequency
Quality	1538	Quality	66
Like	1312	Like	23
Good-looking	1142	Good-looking	22
Comfortable	867	Comfortable	21
Fabric	758	Fabric	20
Production techniques	526	Production techniques	19
Size	446	Size	19
Style	437	Style	17
Fast	420	Fast	16
Color	378	Color	15

In the negative reviews, keywords related to product quality appeared 164 times in negative reviews. The primary factor driving consumer negative evaluations was product quality, followed by merchant services. Whether in positive or negative reviews, “quality” is the most common keyword.

**Fig 14:**
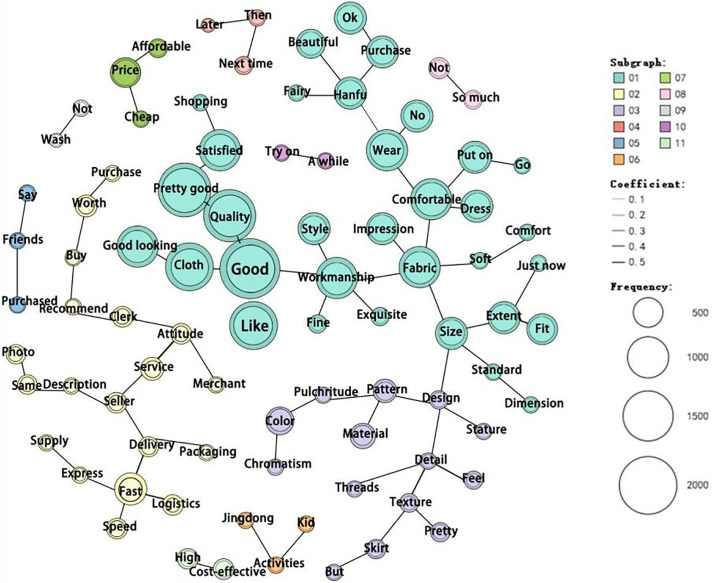
Positive keyword co-occurrence network.

The co-occurrence networks for positive and negative reviews revealed fundamentally different semantic structures. In positive reviews (Fig 14), “quality” co-occurs frequently with “good” “like” and “satisfied.” Similarly, “fabric” frequently co-occurs with “comfortable” and “soft,” while “good” also frequently co-occurs with “matching” and “fit.” This indicates that consumers primarily provide positive feedback on aspects, such as product quality, design, and merchant services. Examples of positive reviews are as follows:

*Consumer 1: “I bought this Hanfu as a birthday gift for my picky sister, and she loves it! The fabric quality is great, the size fits well, and it doesn’t wrinkle. The color is also perfect for her skin tone. The seller provided excellent customer service, and the delivery was timely. Highly recommend!”*

*Consumer 2: “The skirt is beautifully made and very attractive. Although the delivery was delayed, it was worth the wait.”*

*Consumer 3: “The quality exceeded my expectations. The fabric is very comfortable, and there is no color difference. My friend also bought this dress, and she is equally satisfied. It’s very likable, and the delivery time wasn’t long. I suggest buying it in advance.”*

*Consumer 4: “The skirt is very comfortable, and the quality is great. I’m very satisfied, but the top is a bit too big for me.”*

In the positive reviews, some reviews noted that the size was very well-fitting, while others found it difficult to choose the right size, even though they were satisfied with their purchase. This may be because each brand has a sizing system. Therefore, an intuitive size guide is necessary to help consumers choose the right size when shopping online. Additionally, some reviews mention fast delivery, whereas others mention slow delivery. This could be because most modern Hanfu brands adopt a pre-sale model to reduce the financial risks associated with stocks. Therefore, improving the efficiency of order processing is necessary to ensure that consumers receive their purchased products promptly.

**Fig 15:**
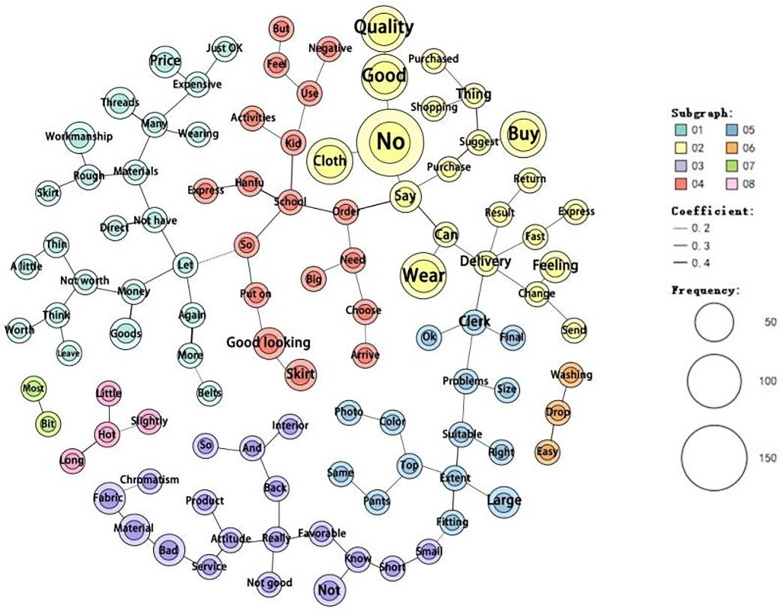
Negative keyword co-occurrence network.

In negative reviews (Fig 15), the co-occurrence frequency of “quality” with “good” and “no” is notably high. Terms such as “poor” “fabric” “material” “service” “attitude” and “color” often appear together. Similarly, “size” “large” “small” and “problem” frequently co-occur, as do “production techniques” “rough” “loose threads” and “too many.” Other commonly associated terms include “not worth” “thin” “money,” “wash” “fade” “easily,” “fast” “delivery” and “courier,” and “beautiful” and “skirt.” These patterns indicate that quality-related complaints were particularly common. Consumer negative evaluations mainly focus on product quality, service, and design. Complaints regarding product quality include poor fabric material, thinness, rough production techniques, excessively loose threads, and garments that easily lose their details after washing. In addition, sizing inconsistencies have been frequently mentioned. Many consumers express dissatisfaction with seller services, citing poor customer service attitudes and discrepancies between product images and actual items received. Issues with product design such as inappropriate garment lengths and thicknesses are common concerns. These aspects highlight the key areas of dissatisfaction among consumers who purchase modern Hanfu. Among these, product quality is the most critical concern, followed by seller service and product design. Examples of negative reviews include the following:

*Consumer 5: “A bit expensive, the quality is not great, and it’s not breathable. But it looks nice. I hope the quality matches the price so I can wear it more often!”*

*Consumer 6: “Looks good, but the quality is just average.”*

*Consumer 7: “The clothes are fine, but the customer service is terrible. Poor attitude, which negatively affects shopping and delivery. I hope customer service can provide genuine assistance rather than robotic responses.”*

*Consumer 8: “The quality is poor, the material is subpar, and the production techniques are mediocre with many loose threads. But the delivery was fast, and the appearance and size were satisfactory.”*

As seen above, while the actual quality of modern Hanfu is not particularly high, most consumers tend to leave positive reviews out of habit. This suggests a significant gap between the actual product quality and the ratings received. Negative reviews focusing on product quality, services, and design provide critical evidence for constructing a scale to measure the factors influencing the online purchase intention for modern Hanfu.

### Grounded theory results

A total of 112 original concepts were analyzed through three-level coding and finally condensed into a theoretical model with four categories and ten themes (Table 7). Among them, the new dimensions such as “website design attractiveness” and “social media participation” validate the findings of e-commerce consumption research and reveal the unique decision-making path of “visual symbol driven——community interaction enhanced” in modern Hanfu consumption. The purchase intention scale constructed based on this framework effectively deconstructs the complex decision-making mechanism in which high cultural value-added and high-quality control coexist and provides a theoretical anchor point for subsequent empirical studies.

**Table 7:** Consumers’ review coding and dimensions generation.

Category	Topic	Example of original encoding	Example of original text
Product information display	Product details	Embroidery/detailed description/premium	“The embroidery is as described, very upscale and good value for the money”
	Visual presentation	Color/color difference/picture	“Very good! Good quality, the same as the physical store, the color is positive, is my favorite” “The same as the picture description, no color difference, wearing is also very good-looking”
Co-design	Brand feedback	Improvement/listening to advise/adoption	“Sleeves improved, quite good-looking, and later with, summer, fall can be worn” “Last year spit sleeves were too big, this year, large sleeves have an ancient feeling and actually will not be inconvenient to move, very good, primarily featuring listening to advice”
	Consumer opinion	Improvement suggestions/style	“Last year said this style is too traditional, changed the style did not think it was quite good, buy good quality, wear good-looking but also cheap”
	Social co-creation	Fellow robe/together	“This shape is a modern Hanfu fellow robe examined the Ming Dynasty cultural relics together after the completion of the”
Website attractiveness	Content presentation	Text/readability/detailed/clear	“The text description on the page is detailed, and the picture is also very clear”
	Color design	Color scheme/harmony/classical/modern	“The home page color scheme is very harmonious, giving a classical and modern combination of feelings”
	Layout	Typography/layout/neat	“The baby page is clear and neat”
Social media engagement	Platform activities	Activity/rednote/activity/community	“Immediately after receiving the small red book, to participate in the recitation of classical and ancient activities is very suitable” “Participate in the national recitation activities to buy, delivery is super fast, the clothes are also very satisfied”
	Interactive behavior	Recorded video/likes/live/interactive	“The child needs to record a video temporary purchase, wear it to be able to achieve the desired effect, completed the recording, many people praise, very happy” “Live when wearing a very warm and gentle gentleman’s model” “Bought for my daughter for a filming event, great value for money”

### Quantitative results

#### Determination of measurement scale.

Based on text analysis, text coding, and a literature review, the nine key factors were as follows: (Table 8): cultural identity, product quality, product uniqueness, merchant service, brand innovativeness, product information display, co-design, website attractiveness, and social media engagement.

**Table 8:** Dimensions influencing consumers’ online purchase intentions for modern Hanfu.

No.	Dimension	Meaning	Sources
1	Cultural Identity	Cultural identity refers to the affirmative recognition of the most significant elements of one’s ethnic group formed by members of a group who have lived together in an ethnic community for a long time. It is a feeling of being influenced by the culture of the group and is a value affirmation of one’s spiritual existence.	Literature review (Hanfu and purchase intention); [5,7,12,28,29]
2	Product Quality	Product quality refers to the sum of the features and characteristics that a product possesses to meet the stipulated and potential needs. Generally, it includes aspects such as suitability, safety, reliability, durability, maintainability, and economy.	Literature review (Hanfu and purchase intention); Text Mining; [67]
3	Product Uniqueness	Product uniqueness refers to the distinctive and differentiated features that a product possesses in functionality, design, material composition, or brand story when compared with other products on the market. Such features can meet consumers’ demands for individuality, novelty, and unique experiences.	Keywords extraction and analysis; [68]
4	Merchant Service	Merchant service refers to a general term for a series of commercial activities and value-added services provided by merchants to meet consumers’ demands, including pre-sale consultation, in-sale transactions, and post-sale support.	Sentiment Analysis of Consumer Reviews; [69–73]
5	Brand Innovativeness	Brand innovation refers to the ability of a brand to create and transform its concepts, products, services, marketing, and other aspects by introducing new thinking, technologies, models, or elements. It enables the brand to demonstrate its unique creativity and transformation capabilities that differ from traditional ones and those of competitors, thereby providing consumers with a brand-new value experience and promoting the continuous development and progress of the brand.	Keywords extraction and analysis; [19,74]
6	Product Information display	Product information display refers to the presentation of key product information through various forms, such as text, pictures, videos, etc., that are clear, comprehensive, and accurate. It includes but is not limited to product features, usage methods, prices, user reviews, and so on to help consumers better understand and evaluate the product, thereby making purchasing decisions.	Consumers’ review coding; [18,61]
7	Co-design	Joint design refers to the process where designers collaborate with users or stakeholders to participate in the design of products, services, or systems, aiming to ensure that the design outcomes meet the needs and expectations of users.	Consumers’ review coding; [75,76]
8	Website attractiveness	The attractiveness of a website page refers to the ability of the page to attract and retain users’ attention through factors such as visual design, content layout, and interactive experience.	Consumers’ review coding; [77–79]
9	Social media engagement	Social media engagement is an important metric for measuring the degree of interaction between users and content on social media platforms. It is usually evaluated through actions such as likes, reviews, and shares.	Literature review (Hanfu and purchase intention) Consumers’ review coding; [19,80–82]

Based on the focus group discussion and analyses by three senior researchers, the resulting evaluation scale comprising 19 statements across six dimensions is presented in Table 9.The consumer purchase intention dimension directly referenced mature measurement scales from the related literature [83–85].

**Table 9:** Consumer purchase intention evaluation scale for modern Hanfu.

Dimension	Code	Item
Cultural identity	CI1	I have a great fondness for the essence of Hanfu
	CI3	I think the culture of Hanfu has enhanced my pride in traditional Chinese culture
	CI4	I hope to see more publicity about the culture of Hanfu
Product quality	PQ1	The size of the product I purchased is just right
	PQ2	The production techniques of the product I purchased are very good
	PQ3	The fabric of the product I purchased is excellent
Merchant service	MS1	Customer service staff can provide me with matching suggestions
	MS2	Customer service staff can continuously improve the quality of service
	MS3	Customer service staff can actively answer my questions
Co-design	CD1	I prefer brands that can offer design suggestions together
	CD2	Participating in product design makes me very happy
	CD3	Participating in product design can enhance my interest in the product
Website attractiveness	WA1	The online page design of this brand is very attractive
	WA2	The online page of this brand has interactivity
	WA3	The information on the online page of this brand is very clear
	WA4	The layout of the online page of this brand is very neat
Social media engagement level	SME2	I have met many people who are interested in modern Hanfu through social media
	SME3	Social media is my main channel to learn about the offline activities of modern Hanfu
	SME4	Recommendations or positive reviews on social media will prompt me to purchase products from this brand
Consumer purchase intention	PI1	I plan to spend more on modern Hanfu rather than conventional clothing
	PI2	I am willing to recommend the brand’s products to others
	PI3	Compared with similar products on the market, I am more inclined to choose the products of this brand

#### Data analysis of the survey.

Among the 344 valid responses collected, there was a clear female majority (n = 251; 73%), and approximately three-quarters of respondents had at least a college degree. Respondents’ ages ranged from 15 to 60 years (mean = 28 years).

The overall Cronbach’s alpha for the 19-item instrument was 0.919, indicating excellent internal consistency. The Kaiser-Meyer-Olkin (KMO) measure was 0.915, and Bartlett’s test of sphericity was significant (χ² = 3932.863, p < 0.001), confirming the suitability of the dataset for factor analysis. The Cronbach’s alpha values for all dimensions were greater than 0.7. Except for the PI dimension, for the remaining dimensions, the composite reliability was greater than 0.7, and the Average Variance Extracted (AVE) was greater than 0.6, indicating that the measurement model had good internal consistency and strong convergent validity [86]. The correlation between the seven latent variables was less than 0.90, and variance inflation factor (VIF) values for all constructs ranged between 1–5, indicating that multicollinearity was not a concern (Table 10) [87]. The square roots of the AVE for each dimension were greater than the correlation coefficients, indicating good discriminant validity among the dimensions (Table 11) [88].

**Table 10:** Reliability and validity test.

Construct	Items	Standardized factor loading	AVE	CR	Alpha	VIF
Cultural identity	CI1	0.755	0.559	0.792	0.728	1.412
	CI3	0.776				
	CI4	0.711				
Product quality	PQ1	0.731	0.550	0.786	0.721	1.443
	PQ2	0.717				
	PQ3	0.776				
Merchant service	MS1	0.783	0.619	0.830	0.775	1.294
	MS2	0.789				
	MS3	0.789				
Joint design	CD1	0.703	0.536	0.776	0.806	1.899
	CD2	0.765				
	CD3	0.727				
Attractiveness of website pages	WA1	0.805	0.570	0.841	0.863	1.819
	WA2	0.728				
	WA3	0.700				
	WA4	0.783				
Social media engagement	SME2	0.816	0.604	0.820	0.847	1.749
	SME3	0.768				
	SME4	0.745				
Consumer purchase intention	PI1	0.643	0.41	0.675	0.882	–
	PI2	0.600				
	PI3	0.676				

**Table 11:** Correlation and discriminant validity.

	SME	WA	CD	MS	PQ	CI	PI
**SME**	*0.777*						
**WA**	0.605	*0.755*					
**CD**	0.695	0.72	*0.732*				
**MS**	0.462	0.436	0.456	*0.787*			
**PQ**	0.423	0.454	0.424	0.455	*0.741*		
**CI**	0.429	0.459	0.44	0.391	0.646	*0.748*	
**PI**	0.677	0.719	0.733	0.569	0.607	0.602	*0.640*

Note(s): The average variance extracted (AVE) are in italic.

#### Structural model path coefficients and fit indices.

**Fig 16:**
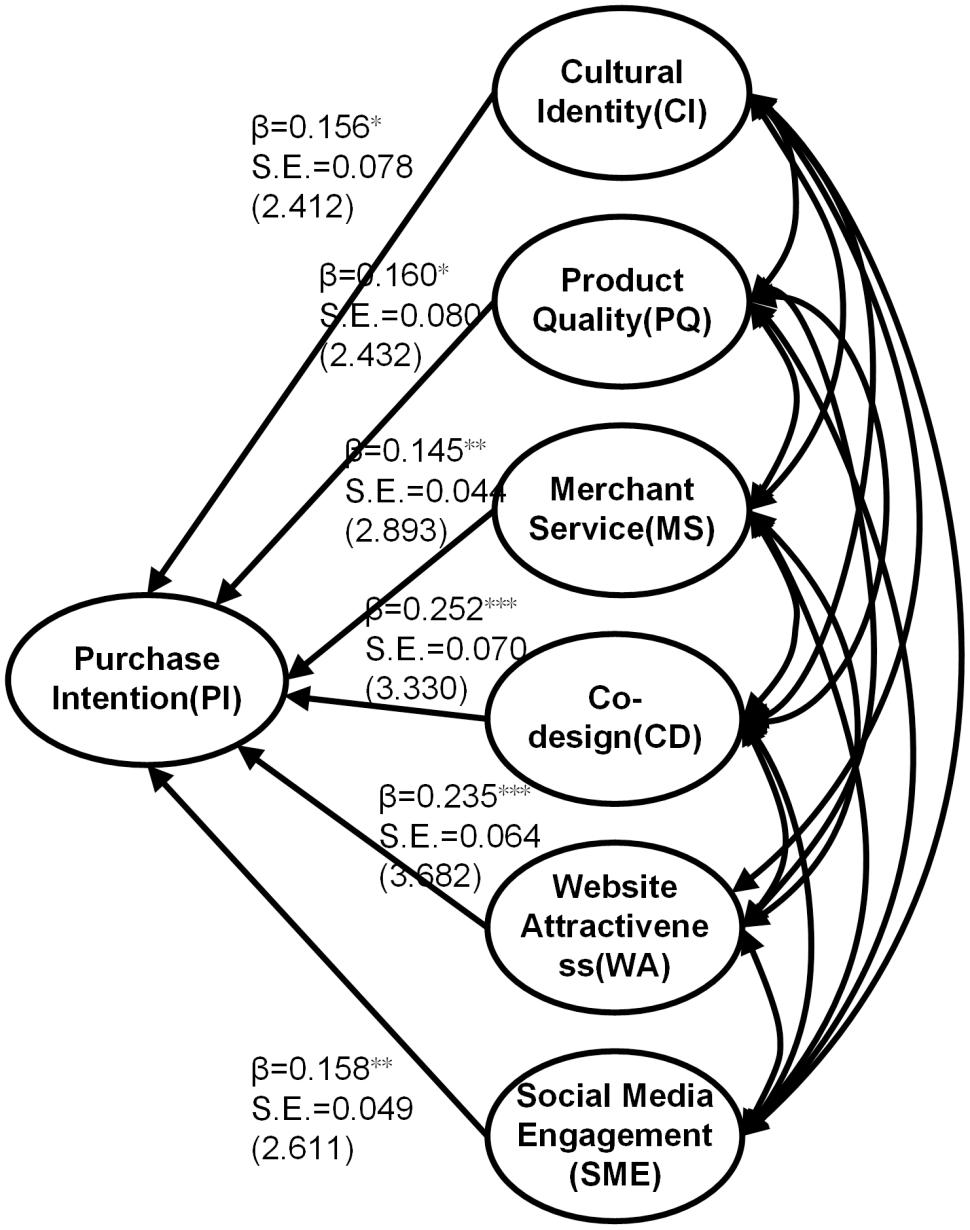
Structural equation model. Note(s): Critical ratios are in parentheses. *p < .05,**p < .01,***p < .001. Abbreviations: S.E., standard error.

The significance of all path coefficients was strong (p < .05; Fig 16). This indicates that each path coefficient was significantly different from zero at the 95% confidence level. The factors of co-design, website attractiveness, and product quality were ranked as the top three influences on consumer purchase intention, with path coefficients of 0.252, 0.235, and 0.160, respectively. The standardized path coefficient for merchant services is 0.145, which is the lowest among all the indicator variables, indicating that it has the least impact on consumer purchase intention.

In the model fit assessment, the key goodness-of-fit indices showed the following results: the χ²/df was 1.479, and the root mean square error of approximation was 0.037, both reaching ideal levels. In addition, other key fit indices passed the tests (Table 12).

**Table 12:** Model fit test results.

Adaptability index	Indicator value	Ideal level	Result
CMIN/DF	1.479	1-8	Acceptable
RMSEA	0.037	<0.08	Acceptable
GFI	0.929	>0.8	Acceptable
IFI	0.977	>0.8	Acceptable
TLI	0.971	>0.8	Acceptable
NFI	0.931	>0.8	Acceptable
CFI	0.976	>0.8	Acceptable

## Discussion and conclusion

Multiple factors significantly influence consumers’ online purchase intention for modern Hanfu. In line with the research objectives, the key findings were as follows:

(1) The text-mining analysis of 5,992 consumer reviews revealed that product quality is the most frequently discussed factor, with keywords such as “fabric” “production techniques” and “comfort” appearing prominently. Consumers consistently praised the quality of materials and paid attention to the details in modern Hanfu designs. Merchant services have emerged as critical factors, with many reviews highlighting the importance of responsive customer support and efficient delivery. However, cultural identity, while present in some reviews, was less frequently discussed, suggesting that although it plays a role in modern Hanfu consumption, it may not be the primary driver of purchase decisions in online reviews.
(2) Study 2 employed grounded theory to systematically construct a theoretical model through two-phase coding analysis. The exploration phase (n = 5,000) was initiated with open coding by a dual-expert team that conducted a semantic unit analysis. For instance, the comment “Last year’s sleeves…primarily featuring listening to advice” yielded concepts like corporate responsiveness and user participation. Axial coding clustered these into meso-themes, notably synthesizing “listening” “improvement” and “adoption” into consumer co-design mechanisms. Selective coding refined the four core dimensions after eliminating redundant categories. The validation phase (n = 992) demonstrated theoretical saturation. The final model divided the 112 concepts into 10 subthemes with four categories (product information display, co-design, website attractiveness, and social media engagement). The dimensions that influence purchase intention operationally define the coexistence of cultural value creation and quality control mechanisms, thereby establishing a conceptual foundation for subsequent empirical verification.
(3) A quantitative analysis based on a survey of 344 modern Hanfu consumers confirmed and extended the findings of Study 1. Co-design exhibited the strongest impact on purchase intention (β = 0.252), followed by website attractiveness (β = 0.235) and product quality (β = 0.160). Social media, while significant, had a moderate influence (β = 0.158), and cultural identity was a significant but less dominant factor (β = 0.156). Merchant service, despite its potential for enhancing consumer engagement, showed the weakest effect (β = 0.145), indicating that consumers do not prioritize the quality of service provided by customer service agents when purchasing modern Hanfu.
(4) While Study 1 highlights product quality as the most frequently discussed factor in consumer reviews, Study 3 reveals that co-design has the strongest influence on purchase intentions. This discrepancy can be attributed to the different methodologies used in these two studies. Text mining captures the explicit concerns and preferences expressed by consumers in their reviews, which tend to focus on tangible aspects, such as product quality and merchant service. By contrast, the quantitative analysis in Study 3 measures the relative strength of various factors in predicting purchase intention, revealing that intangible factors such as co-design and website attractiveness play a more significant role in shaping consumer decisions. This suggests that while consumers may prioritize product quality in their reviews, their actual purchase decisions are more strongly influenced by co-design and website attractiveness.

These findings are consistent with prior research that highlighted the importance of co-design in shaping consumer behavior [89–91], particularly in the fashion sector. Consumer participation in modern Hanfu design solves the pain points of traditional modern Hanfu, such as “cumbersome wearing” and “single scene,” and upgrades modern Hanfu from a “minority interest” to a “popular trend” through emotional connection and cultural co-creation. This plays a crucial role in the pursuit of personalized expression in young modern Hanfu consumers who have special needs for styles, patterns, materials, and so on. Similarly, website attractiveness resonates with Majeed et al. [77] and others, who emphasized the importance of a brand’s website design in e-commerce, where the attractiveness of the website page, its layout, clarity of information on the page, and interactivity have a significant impact on consumers’ willingness to buy, with the only difference that this study does not consider 360 degrees and virtual “try-on” separately, as these features are mandatory on popular shopping websites.

However, the relatively modest impact of product quality aligns with postmodern consumer theories, which emphasize emotional, symbolic, and identity-based drivers over purely functional evaluations [67]. Although product quality appears frequently in consumer reviews, such mentions may reflect post-purchase satisfaction rather than pre-purchase intention. In contrast, co-design fosters emotional attachment and cultural alignment, which are stronger predictors of purchase intention in symbolic fashion contexts [92,93]. This difference can be attributed to the unique cultural and emotional appeal of modern Hanfu. In modern Hanfu consumption, consumer aesthetic value, cultural identity, and sense of belonging to a cultural movement often outweigh their concerns about product functionality. Cultural identity has a positive effect on consumer willingness to purchase products with the corresponding cultural value attributes. This finding aligns with Chakraborty [5], who argue that cultural values significantly influence sustainable fashion consumption, and extend this framework by linking cultural identity specifically to modernized traditional attire. In addition, social media provides the opportunity to build emotional connections and create a sense of community, which is an important factor influencing young consumers’ purchase intentions [29,81].

Further, while customer service and post-purchase support are important in e-commerce, their relatively weak effect in this study merits a more nuanced interpretation. One possible explanation is that modern Hanfu consumers have become highly familiar with product categories, sizing, and seller practices, reducing their reliance on active service interactions. In addition, many e-commerce platforms now rely on automated or standardized service mechanisms—such as AI-based chatbots, detailed product pages, and self-service return systems—which fulfill baseline expectations but no longer constitute a major influence on purchase decisions. More importantly, in identity-rich product categories like modern Hanfu, symbolic expression and cultural resonance often outweigh utilitarian concerns. Consumers may thus prioritize co-design, aesthetic value, and community alignment over responsiveness or delivery efficiency. This supports a broader shift from utility-driven to identity-anchored consumption in fashion subcultures, especially within digital-native consumer groups.

### Implications and limitations

This study has important theoretical and management implications. Theoretically, this enriches the existing purchase intention model by incorporating cultural identity and co-design as novel factors in heritage-driven fashion consumption. This framework broadens our understanding of the interplay between consumer behavior and cultural heritage, offering a more nuanced perspective on the factors that drive purchase decisions in niche, culturally significant markets such as modern Hanfu. This study contributes to the literature in three ways.

(1) Integration of cultural identity into purchase intention models. While traditional models such as the TPB and SOR focus on psychological and environmental factors, this study demonstrates that cultural identity plays a critical role in shaping purchase intentions for heritage-driven products, such as modern Hanfu.
(2) The finding that co-design is a key driver of purchase intention reflects the shift in the role of consumers from “cultural spectators” to “cultural co-creators” and provides a direction for the transformation of the modern Hanfu industry, as well as a reference path for the modernization of other traditional garments. Additionally, this study enriches the discourse on cultural product consumption by integrating co-design into the framework of cultural identity and participatory value creation. This suggests that co-design transcends mere customization; it acts as a cultural engagement tool that bridges traditional aesthetics with contemporary consumer demands for self-expression and cultural authenticity. This aligns with, and extends, the concept of “cultural co-creation,” where co-design processes empower consumers to reinterpret heritage symbols (e.g., modern Hanfu patterns and silhouettes) through a modern lens, thereby reinforcing emotional resonance and cultural belonging, a dimension under exploration in the existing literature on online apparel consumption.
(3) Customer service has been traditionally reassessed. In traditional fashion, consumers pay more attention to core elements such as product design, product quality, and cultural attributes when purchasing modern Hanfu than to the service experience in traditional retailing. This phenomenon may stem from the specificity of modern Hanfu consumption; consumers tend to view modern Hanfu as a cultural symbol or a tool for personalized expression, rather than as ordinary clothing. This implies that a handful of brands need to position service as a supplementary function, rather than as a core competency. This finding challenges the common assumption that customer service is key in traditional retailing and requires brands to reassess their resource allocation; they need to invest more in cultural narratives and product innovation rather than over-optimizing service details.

In addition to incorporating co-design and cultural identity, this study refines the structural logic of established models such as TPB and SOR by recontextualizing them within culturally symbolic consumption. Rather than adopting their original constructs wholesale, we propose a culturally anchored framework where co-design acts as a participatory emotional trigger (Organism) and cultural identity represents a symbolic internalized driver. These mechanisms are rarely addressed in traditional applications of TPB/SOR, which often overlook symbolic, aesthetic, and heritage-based motivations. By identifying how identity-anchored factors outweigh utilitarian ones, this study contributes to a theoretical shift toward value systems driven by emotional resonance, symbolic participation, and digital co-creation—dimensions critical to modern Hanfu and potentially generalizable to other high-identity product categories.

Managerially, the findings of this study have insightful implications for practitioners in the modern Hanfu industry in their endeavors to encourage consumer engagement and boost sales.

(1) Reconstructing a consumer participation mechanism using collaborative design as the core. Given the critical impact of collaborative design on consumer purchasing intentions, modern Hanfu brands can build online interactive platforms that allow consumers to vote for patterns and colors or participate in virtual try-ons to enhance their sense of participation. For example, short-video platforms such as TikTok can collect user preferences through live interaction, forming a closed loop of “design-feedback-optimization,” which can strengthen the interaction and emotional connection with the brand and increase the willingness to buy.
(2) Strengthen website attractiveness and optimize digital reach paths. As website attractiveness is a key determinant, brands are required to redefine online interaction scenarios, such as displaying the dynamic effects of modern Hanfu through short videos and live broadcasts; using algorithmic recommendations on platforms such as TikTok and Bilibili to accurately reach young users; and optimizing e-commerce page design by adding 3D try-on and AR accessory matching functions, which can lower the threshold of consumer decision-making on sizes, fits, patterns, textures, color matches, and materials and can give consumers more confidence in their purchasing decisions, especially in online environments where they cannot try products in person.
(3) Enhancing product quality standards and balancing cultural attributes with practicality. Despite the importance of the cultural and emotional connections represented by modern Hanfu, the assurance of product quality still plays an important role in promoting willingness to buy and is the basis for building long-term brand loyalty. Modern Hanfu brands should emphasize the optimization and upgrading of materials and processes, such as using silk, cotton linen, and other traditional fabrics combined with modern technology to improve fabric antibacterial and anti-wrinkle treatment capabilities and enhance functionality and differentiation. Concurrently, brands should focus on daily design optimization by addressing consumer feedback on inconvenience (e.g., putting on or taking off modern Hanfu), simplifying the wearing structure (removable accessories, elastic waist seals), and introducing seasonal adaptations (breathable tulle models in the summer) to meet consumers’ needs for both “practical value” and “spiritual value.”

This study has some limitations. First, the sample is predominantly composed of female consumers (73%) and younger consumers (mean age = 28 years), which may limit the generalizability of the findings across different age groups or male consumers. Future studies should explore sex- and age-related differences in the intention to purchase modern Hanfu. Additionally, this study relies on reviews from two Chinese e-commerce platforms (Taobao and JD), which may not fully capture global consumer sentiment or preferences in offline retail settings. This limitation could be addressed in future research by expanding the geographical scope to include offline consumer perspectives.

Second, the cross-sectional design of this study limits the ability to make causal inferences about the relationship between the identified factors and purchase intention. Longitudinal studies would provide more insight into how consumer preferences evolve, particularly as modern Hanfu transitions from a niche to a mainstream market. Moreover, the self-reported nature of the data may introduce bias, as consumer responses may not always reflect actual purchasing behaviors.

Future research could explore the role of demographic diversity (e.g., male consumers and older generations) in shaping modern Hanfu purchase intentions, as these groups may have different motivations and purchasing behaviors. Comparative studies across cultural contexts such as overseas Chinese communities would deepen our understanding of how globalization affects the consumption of heritage apparel. Further, integrating advanced techniques, such as machine learning and natural language processing, to analyze multimodal data (e.g., video reviews and social media posts) could uncover additional latent drivers of purchase behavior.

Finally, experimental designs can be used to test the efficacy of co-designed platforms in enhancing consumer engagement and loyalty. As modern Hanfu brands incorporate consumer feedback and participatory design features, it is valuable to evaluate the impact of these initiatives on brand perception and purchase intention.

## Supporting information

S1 File Consumer Review Collection. This file contains 5,992 consumer online reviews collected from nine brands (A–I) across two major e-commerce platforms. These reviews were used as the primary data source for the text-mining analysis conducted in Study 1.
(XLSX)

S2 File Python SNOWNLP Code. This file provides the Python code based on the SnowNLP library. It was used to perform sentiment analysis on the consumer review dataset.
(TXT)

S3 File Survey questionnaire raw data. This file includes the raw data obtained from the survey. The dataset was used for the empirical analysis of the consumer purchase intention evaluation scale.
(XLSX)
